# BRCA1 tumours correlate with a HIF-1*α* phenotype and have a poor prognosis through modulation of hydroxylase enzyme profile expression

**DOI:** 10.1038/sj.bjc.6605287

**Published:** 2009-09-01

**Authors:** M Yan, M Rayoo, E A Takano, H Thorne, S B Fox

**Affiliations:** 1 Department of Pathology, Peter MacCallum Cancer Centre, St Andrews Place, East Melbourne, Victoria 3002, Australia;; 2 KconFab, Peter MacCallum Cancer Centre, St Andrews Place, East Melbourne, Victoria 3002, Australia

**Keywords:** HIF-1α, FIH, breast, BRCA1, familial

## Abstract

**Background:**

There are limited data regarding the hypoxia pathway in familial breast cancers. We therefore performed a study of hypoxic factors in BRCA1, BRCA2 and BRCAX breast cancers.

**Methods:**

Immunoperoxidase staining for HIF-1*α*, PHD1, PHD2, PHD3, VEGF and FIH was carried out in 125 (38 BRCA1, 33 BRCA2 and 54 BRCAX) breast carcinomas. These were correlated with clinicopathological parameters and the intrinsic breast cancer phenotypes.

**Results:**

BRCA1 tumours correlated with positivity for HIF-1*α* (*P*=0.008) and negativity for PHD3 (*P*=0.037). HIF-1*α* positivity (*P*=0.001), PHD3 negativity (*P*=0.037) and nuclear FIH negativity (*P*=0.011) was associated with basal phenotype. HIF-1*α* expression correlated with high tumour grade (*P*=0.009), negative oestrogen receptor (ER) status (*P*=0.001) and the absence of lymph node metastasis (*P*=0.028). Nuclear FIH expression and PHD3 correlated with positive ER expression (*P*=0.024 and *P*=0.035, respectively). BRCA1 cancers with positive HIF-1*α* or cytoplasmic FIH had a significantly shorter relapse-free survival (*P*=0.007 and *P*=0.049, respectively).

**Conclusions:**

The aggressive nature of BRCA1 and basal-type tumours may be partly explained by an enhanced hypoxic drive and hypoxia driven ER degradation because of suppressed PHD and aberrantly located FIH expression. This may have important implications, as these tumours may respond to compounds directed against HIF-1*α* or its downstream targets.

Carriers of germline mutations in BRCA1 and BRCA2 have a predisposition for developing breast and/or ovarian cancer. The cumulative risk of breast carcinoma in carriers of BRCA1/BRCA2 mutations ranges from 45–84% by 70 years of age (Antoniou *et al*, 2003). There are chromosomal, morphological and immunohistochemical differences between spontaneous and BRCA-associated tumour (Lakhani *et al*, 2000; Jonsson *et al*, 2005; Palacios *et al*, 2005). Thus BRCA1 mutation associated breast cancers are generally poorly differentiated and more frequently display typical and atypical medullary-like morphology than sporadic tumours (Lakhani *et al*, 2000). Furthermore, BRCA1 tumours show a so-called ‘triple negative’ phenotype being oestrogen receptor (ER), progesterone receptor (PgR) and HER2 negative (Palacios *et al*, 2003). BRCA1 tumours also harbour p53 mutations (Sensi *et al*, 2003) and express basal and myoepithelial markers such as cytokeratins (CK) 5, CK14, *α*-actin and p63 in keeping with a basal-like phenotype (Jacquemier *et al*, 2005; Laakso *et al*, 2005; Lakhani *et al*, 2005). Although reports suggesting lobular carcinomas are more frequent in BRCA2 carriers, no similarly defined phenotype has been described for BRCA2-associated tumours, which usually show a ductal, no special type morphology and ER positivity (Armes *et al*, 1998). These observations suggest specific and distinct neoplastic pathways in germline BRCA mutation-related breast cancers compared with sporadic cancer pathways.

Hypoxia is a hallmark of many cancers that has been associated with diminished therapeutic response and with an increasing probability of malignant progression (Vaupel, 2004). Hypoxia-inducible factor-1 (HIF-1) is the key regulator of the hypoxia response. HIF-1 consists of two subunits, HIF-1*α* and HIF-1*β*. Although HIF-1*β* is a nuclear protein that is constitutively expressed and is independent of oxygen tension, the HIF-1*α* protein is induced and continuously degraded under normoxia by the ubiquitin–proteasome pathway in the cytoplasmic cellular compartment (Salceda and Caro, 1997; Huang *et al*, 1998). However, under hypoxic conditions HIF-1*α* translocates to the nucleus where it heterodimerizes with HIF-1*β* (Vaupel, 2004). This HIF-1 complex then regulates the expression of its target genes through binding with hypoxia responsive elements in the promoter regions of target genes (Bos *et al*, 2003; van der Groep *et al*, 2008) including erythropoietin, vascular endothelial growth factor (VEGF), glycolytic enzymes, transferrin and a variety of other proteins that enhance tumour survival, invasion and metastasis (Semenza, 2000; Vaupel, 2004).

The level and activity of HIF-1*α* subunit is tightly regulated through a number of post-translational modifications. In the presence of oxygen, three prolyl hydroxylase enzymes (PHD), PHD1, PHD2 and PHD3 cause site-specific hydroxylation of two proline residues, P402 and P564, within the oxygen-dependent degradation domain of HIF-1*α*. This allows for the recognition of HIF-1*α* by the tumour suppressor von Hippel-Lindau protein, which targets HIF-1*α* for degradation (Boddy *et al*, 2005). In addition, hydroxylation of the asparagine residue 803 by factor inhibiting HIF (FIH) reduces its transcriptional activity through interfering with cofactor binding (Lando *et al*, 2002). However, under hypoxic conditions these hydroxylase enzymes are less active because of the paucity of molecular oxygen, resulting in both an increase in the level of nuclear HIF-1*α* and its activity (Boddy *et al*, 2005).

In sporadic breast cancer, previous studies have shown that HIF-1*α* overexpression has a role in breast carcinogenesis and is correlated with a poor prognosis (Generali *et al*, 2006). However, little is known about the role of HIF-1*α* in hereditary breast carcinogenesis with only one report in a small series (*n*=30) suggesting overexpression of HIF-1*α* present in a higher frequency in BRCA1-related cancers than sporadic cancers (van der Groep *et al*, 2008). Given the importance of all pathway members in the control of hypoxia-induced gene expression, our aims were to: (1) determine the level and pattern of expression of HIF-1*α*, PHDs and FIH in a large cohort of familial breast cancers, (2) correlate expression with conventional clinicopathological parameters, (3) investigate expression in familial breast cancers stratified by intrinsic breast cancer phenotypes and (4) explore their role in patient survival.

## Materials and methods

### Patients and tumour tissue microarrays

Tumour tissue microarray cores (1 mm cores) with fourfold redundancy for 147 familial invasive breast cancers were collected from the kConFab biorepository. For the purposes of this study, classification of BRCA1 and BRCA2 mutations and sequence variants was according to designations listed for research purposes on the kConFab website (www.kconfab.org/index.shtml). The BRCAX breast cancers are defined by familial breast cancer in families without a known BRCA1 and BRCA2 pathogenic mutation. General inclusion criteria for the BRCAX subgroup were families with breast cancer meeting kConFab category 1 and 1B eligibility criteria and with a breast cancer pathology report held by kConFab.

The flow of patients through the study according to the REMARK criteria is listed in Table 1 (McShane *et al*, 2005). Of the 147 cases, 9 cases were excluded because of the lack of tissue available for array construction and a further 13 cases were excluded because of the absence of tumour on the array available for staining. The final cohort was composed of 125 cases composed of 38BRCA1, 33BRCA2 and 54 BRCAX cases.

These were compared with a cohort of 186 sporadic cancers collected from the John Radcliffe Hospital, Oxford, UK, which was characterised in a previous study (Tan, 2008). All patients had operable breast carcinomas and were not diagnosed with metastatic disease at the time of presentation. Information regarding patient characteristics, including age, tumour size, grade, histology, nodal status, ER and HER-2 status were collected from the clinical and pathological records (Table 2). Using stratification of intrinsic phenotypes based on Nielsen *et al* (2004) tumours were placed into luminal (ER*α* positive, HER2 negative, cytokeratin (CK) 5/6 and/or EGFR negative or positive), basal (HER2 and ER*α* negative; CK5/6 and/or EGFR positive), HER2 (HER2 positive, ER*α*, CK5/6 and EGFR negative or positive) and null/negative (HER2, ER*α*, CK5/6 and EGFR negative).

Patients less than 50 years of age with node positive, ER-negative tumours or tumours larger than 3 cm received adjuvant chemotherapy. Patients with hormone responsive tumours who were more than 50 years of age received 5 years of endocrine therapy. Patients were followed up for a median period of 64 months. During this time, 38 patients relapsed and 31 died (the recorded deaths were breast cancer related otherwise were censored).

### Immunohistochemistry

TMA sections were cut from each block at 4 *μ*m thick intervals, dewaxed, placed through graded alcohol and placed into water. Antigen retrieval was performed in PT Link (Dako, Glostrup, Denmark) using low pH for PHD1, PHD2 and PHD3 and high pH for HIF-1*α*, EnVision FLEX Target Retrieval Solution (Dako) for 20 min at 100°C. VEGF required antigen retrieval in pH 8 buffer (20 mM Tris/1 mM EDTA/10 mM sodium citrate) for 2 min in a pressure cooker. Endogenous peroxidase was blocked with EnVision FLEX Peroxidase-Blocking Reagent (Dako) before incubating the sections with respective monoclonal antibodies. PHD1 (112), PHD2 (366G/76), PHD3 (EG188e) and FIH (162c) antibodies were kindly donated by Professor Kevin Gatter, the Nuffield Department, Clinical Laboratory Sciences, John Radcliffe Hospital, Soilleux *et al*, 2005. Antibodies for VEGF and HIF-1*α* were purchased from NeoMarkers (Fremont, CA, USA) and BD Transduction Laboratories (Lexington, KY, USA). Antibodies were used at the following concentrations: Neat supernatant for overnight at room temperature for PHD1, PHD2 and PHD3, 1 : 50 for 30 min at room temperature for FIH, 1 : 200 for 30 min at room temperature for VEGF and 1 : 50 overnight at 4°C for HIF-1*α*. Antigen–antibody complex was detected using Envision FLEX system (EnVision FLEX/HRP and EnVision FLEX DAB+ Chromogen, Dako). All the slides were counterstained with hematoxylin subsequently; they were dehydrated, cleared and mounted for the assessment.

### Scoring criteria and cutoffs

Scoring was done according to a previously used semi-quantitative system (Boddy *et al*, 2005; Soilleux *et al*, 2005; Tan *et al*, 2007; Couvelard *et al*, 2008). HIF-1*α* was scored only according to the presence (1+) or absence (0) of nuclear expression. For FIH, and all PHDs (both nuclear and cytoplasmic) and VEGF (cytoplasmic only), the intensity was scored as follows: 0, negative; 1, weak; 2, moderate and 3, strong staining. Using a previous defined cutoff which separates the cohort into approximately two groups of equal numbers (Tan *et al*, 2007, 2008), tumours were considered positive if >10% of tumour cells showed staining at equal or more than moderate staining. The same cutoff was used for VEGF, as previously defined (Martin *et al*, 2007; Noike *et al*, 2008).

### Statistical analysis

Comparisons were made using either the one-way ANOVA, log rank or *χ*^2^-test where appropriate. Kaplan–Meier survival curves were calculated using tumour recurrence (defined as the first re-appearance of tumour at any site following definitive treatment) and cancer-related death as the end points and compared using a log-rank test. Binary logistic regression was used for multivariate analyses and the Cox proportional hazard regression model was used to identify independent prognostic factors for disease-free and overall survival. Analyses were performed with SPSS 16.0 (SPSS Inc., Chicago, IL, USA). A two-tailed *P*-value test was used in all analyses and a *P*-value of less than 0.05 was considered statistically significant.

## Results

### Expression of hypoxia pathway members in familial breast cancers

HIF-1*α* showed predominantly nuclear expression in tumour cells, whereas PHDs and FIH showed both nuclear and cytoplasmic staining as previously reported (Boddy *et al*, 2005; Soilleux *et al*, 2005). Staining for VEGF occurred exclusively in the cytoplasm. The range of expression was variable with HIF-1*α* ranging from heterogeneous weak to strong nuclear staining, whereas all the PHDs, VEGF and FIH generally displayed more homogenous staining although this was of variable intensity (Supplementary Figure 1).

When stratified by BRCA mutation status, HIF-1*α* positivity was significantly correlated with BRCA1 (23/32, 72%) compared with BRCA2 (12/32, 38%) and BRCAX (20/49, 41%) associated tumours (*P*=0.008) (Table 3). Cytoplasmic PHD3 positivity was significantly associated with BRCA2 (23/33, 70%) and BRCAX (39/54, 72%) compared with BRCA1 (18/38, 47%) (*P*=0.037) tumours, but there was no significant difference in expression of cytoplasmic PHD1 and PHD2 within the familial breast cancer groups (*P*=0.105 and *P*=0.615, respectively) (Table 3). BRCA1 tumours were more likely to be negative for nuclear FIH, although this did not reach statistical significance (*P*=0.062). VEGF, nuclear PHDs and cytoplasmic FIH showed no significant difference between the familial groups (*P*>0.05).

When familial tumours were stratified by intrinsic phenotypes based on Nielsen *et al* (2004), cytoplasmic PHD3 negativity was significantly associated with a basal phenotype (19/37, 51%) compared with luminal phenotype (39/49, 80%) (*P*=0.037) (Table 4a). Similarly, nuclear FIH negativity was also associated with basal phenotype (30/36, 83%) compared with luminal phenotype (25/46, 59%) (*P*=0.011) (Table 4b). In contrast, HIF-1*α* positivity was significantly associated with basal (27/36, 75%) compared with luminal phenotype (14/47, 30%) (*P*=0.001) (Table 4c). Other hypoxia pathway members examined showed no significant differences in expression between the phenotypes (*P*>0.05).

### Expression of hypoxia factors between familial and sporadic breast cancers

The expression of cytoplasmic PHD2 (106/121, 88%), cytoplasmic PHD3 (80/125, 64%) and VEGF (101/119, 84%) was significantly associated with familial breast cancers as a combined group compared with sporadic cancers (cytoplasmic PHD2 (65/165, 39%), cytoplasmic PHD3 (65/165, 39%), VEGF (92/182, 51%)) (all *P*<0.001). In contrast both nuclear and cytoplasmic FIH expression were significantly correlated with sporadic (133/179, 74 and 114/179, 64%) compared with familial cancers (62/118, 53 and 39/118, 33%) (both *P*<0.001). There was no significant difference in HIF-1*α*, cytoplasmic PHD1 and nuclear PHD1-3 expression between familial and sporadic cancers (both *P*>0.05).

### Correlation of hypoxia factors with clinicopathological parameters in familial breast cancers

HIF-1*α* expression correlated with high tumour grade (*P*=0.009), negative oestrogen receptor-*α* (ER) status (*P*=0.001) and the absence of lymph node metastasis (*P*=0.028) (Table 5). On multivariate analysis using binary logistic regression, including grade, size, lymph node, HER2 and ER status, only negative ER significantly correlated with HIF-1*α* expression (*P*=0.039, Hazard ratio=0.289, 95% CI for hazard ratio 0.089–0.941).

Cytoplasmic FIH expression correlated with positive ER (*P*=0.029) and lower tumour grade (*P*=0.036) (Table 5). The correlation between cytoplasmic FIH expression and lower grade was confirmed on multivariate analysis including size, lymph node, HER2 and ER status (*P*=0.024, Hazard ratio=0.273 and 95% CI for hazard ratio 0.089–0.840). Nuclear FIH also correlated with positive ER (*P*=0.024).

Cytoplasmic PHD1 correlated with tumour size (*P*=0.008), whereas cytoplasmic PHD3 correlated with tumour size (*P*=0.037) and ER expression (*P*=0.035). Cytoplasmic PHD2, nuclear PHD1-3 and VEGF did not show any significant association with any of the clinicopathological parameters.

### Survival analysis in familial breast cancers

When familial cancers were assessed as a combined group, there was no significant correlation in relapse-free or overall survival with hypoxia factors HIF-1*α*, FIH, VEGF and all PHDs (*P*>0.05).

**Figure 1:**
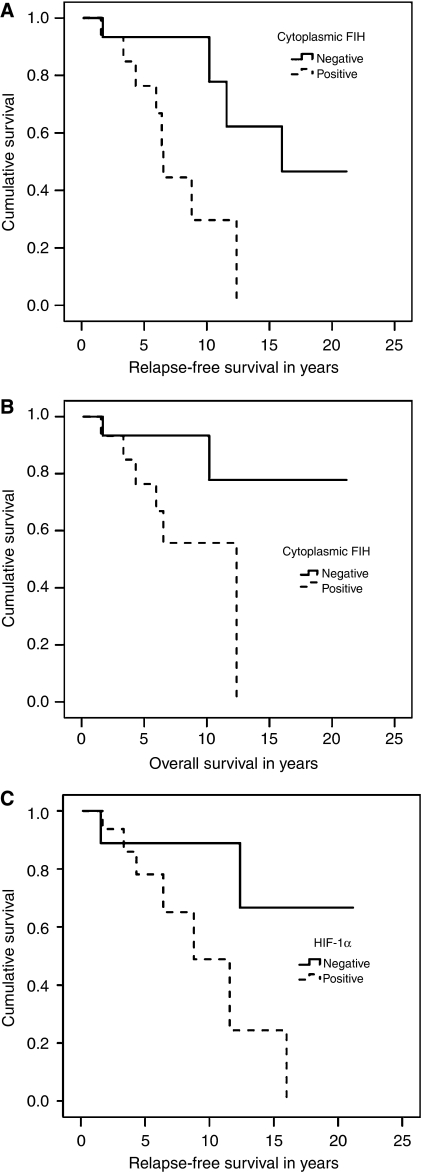
(**A**) Kaplan–Meier curve of relapse-free survival of BRCA1 tumours stratified by cytoplasmic FIH (*P*=0.007, *n*=35). (**B**) Kaplan–Meier curve of overall survival of BRCA1 tumours stratified by cytoplasmic FIH (*P*=0.026, *n*=35). (**C**) Kaplan–Meier curve of relapse-free survival of BRCA1 tumours stratified by HIF-1*α* (*P*=0.049, *n*=31).

However, when BRCA1 cancers were assessed as a separate group, cytoplasmic FIH correlated with shorter relapse-free (*P*=0.007) (Figure 1A) and overall survival (*P*=0.026) (Figure 1B). No such differences in disease-free or overall survival were seen for nuclear FIH (*P*>0.05). BRCA1 tumours with HIF-1*α* expression was associated with a shorter relapse-free (*P*=0.049) (Figure 1C) but not overall survival (*P*=0.764). There was a trend for cytoplasmic PHD1-positive tumours with BRCAX mutation to be associated with a shorter relapse-free survival, although this did not reach statistical significance (*P*=0.058). For BRCA2 tumours there was no significant correlation present between survival and expression of hypoxia-induced factors (*P*>0.05).

## Discussion

Hypoxia is the result of an imbalance between oxygen delivery and oxygen consumption resulting in the reduction of oxygen tension below the normal level for a specific tissue (Lundgren *et al*, 2007). Hypoxia occurs in many disease processes, and it is widespread in solid tumours because of the tumour outgrowing the existing vasculature. Hypoxia is associated with aggressive behaviour, metastasis and lower survival (Harris, 2002). Central to the hypoxic response is the transcription factor HIF. We have therefore examined the role of the key pathway members regulating HIF-mediated transcription including HIF-1*α*, PHD1, PHD2, PHD3, VEGF and FIH in a series of familial breast cancers stratified by BRCA status and intrinsic phenotypes.

In this study, we have shown frequent expression of HIF-1*α* in the neoplastic cells of BRCA1-related breast cancer and basal-like cancers (72 and 75%, respectively) of tumours being positive. This is in accordance with 90% positivity in a small series of 30 patients with BRCA1 mutation (van der Groep *et al*, 2008) and our previous findings where basal-like breast cancers have an enhanced hypoxic drive and aggressive behaviour (Tan, 2008). The observation in this study of a positive association of HIF-1*α* with high tumour grade and relapse-free survival supports a similarly upregulated hypoxic response in BRCA1/basal like cancers. Furthermore, it may also partly account for the increased genomic instability described in BRCA1-associated tumours (Cheng and Loeb, 1993; Sutherland, 1998; Semenza, 2000; Chan *et al*, 2008).

Our findings also support the role of hypoxia and HIF-1*α* in the downregulation of ER*α* expression in familial cancers. In a multivariate analysis, HIF-1*α* was the only independent variable, which predicted ER*α* negativity. This is consistent with cell culture studies where intermittent hypoxia induces proteasome-dependent downregulation of ER*α* (Kurebayashi *et al*, 2001; Cooper *et al*, 2004; Yi *et al*, 2009). This is also supported by immunohistochemical studies on ER*α*-positive tumours where the geographic distribution of CAIX, a surrogate marker of hypoxia, corresponds to areas of tumour negative for ER*α* (Cooper *et al*, 2004). This sequence may enhance the proposed role of BRCA1 in regulating ER*α* expression in BRCA1 and basal like cancers (Gorski *et al*, 2009).

One potential mechanism of retaining elevated HIF-1*α* in both BRCA1 and basal-like may be the relative paucity of PHD3, which showed significantly lower expression in BRCA1 tumours. Under normal conditions, BRCA1 interacts with STAT1 to activate the transcription of IFN-*γ* target genes (Ouchi *et al*, 2000). In the hypoxic state, this pathway selectively induces PHD3, but not PHD1 or PHD2 mRNA and protein expression (Gerber *et al*, 2009). Thus, the loss of PHD3 expression induced by the BRCA1/STAT dysfunction may be a mechanism specific to BRCA1 tumours. Furthermore, hypoxia in basal type/BRCA1 tumours may lead to the preferential degradation of PHD3. The lack of an N-terminal extension in PHD3, which is found in PHD1 and PHD2, renders it more susceptible to degradation by RING finger E3 ligase Siah2 in low oxygen tensions (Nakayama *et al*, 2007). The absence of a prolyl hyrdoxylase may allow the escape of HIF degradation even in the presence of molecular oxygen. This is because significant effects on HIF occur even when the impact of suppression of a single PHD on the total level of PHD protein is only modest (Appelhoff *et al*, 2004). Indeed, PHD3 has been shown to have major effects on HIF, with its loss substantially prolonging the half-life of HIF-1*α* (Appelhoff *et al*, 2004).

In the normoxic state PHD2 is the main cellular oxygen sensor (Berra *et al*, 2003; Berchner-Pfannschmidt *et al*, 2008). Berra *et al* (2003) showed that under normoxic conditions, siRNA inhibition of PHD2, but not PHD1 or PHD3 resulted in the stabilisation of HIF-1*α*. Interestingly, the same author found PHD2 was upregulated by hypoxia, suggesting changes in PHD2 levels were not responsible for HIF-1*α* stabilisation under low oxygen tensions. This is supported by our study of BRCA1 tumours, where upregulation of HIF-1*α* was not associated with changes in PHD2 expression.

In the present study, the intracellular location of FIH appears to have an important role in HIF-1*α* regulation and prognosis, with BRCA1 and familial basal type cancers being negative for nuclear FIH and positive for HIF-1*α*. In addition, cytoplasmic FIH appears as a prognostic marker in BRCA1 breast tumours conferring a shorter relapse-free and overall survival, despite its association with a lower grade, whereas no such differences are seen for nuclear FIH. This is in keeping with our previous studies where cytoplasmic FIH expression correlated with a poorer prognosis (Tan *et al*, 2007). These findings do not appear to be due to nuclear staining being more difficult to appreciate in the presence of strong cytoplasmic staining, as there was a positive correlation between nuclear and cytoplasmic FIH (Spearman *r*=0.318, *P*<0.001). FIH inhibits HIF-1*α* activity by hydroxylating the asparagine residue 803, thereby preventing HIF-1*α* from interacting with co-factor p300 (Lando *et al*, 2002). This effect may be exclusively mediated by FIH located in the nucleus. Translocation of FIH into the cytoplasm may be a mechanism by which BRCA1 and basal-type tumours escape inhibition of HIF-1*α* activity.

Our findings suggest the aggressive nature of BRCA1, basal-type tumours may be partly explained by an enhanced hypoxic drive and hypoxia-driven ER degradation, due to suppressed PHD and aberrantly located FIH expression. This may have important treatment implications, as these aggressive tumours may respond to compounds directed against HIF-1*α* or its downstream targets (Lundgren *et al*, 2007; Milani and Harris, 2008). Of particular interest include agents targeting FIH because, unlike the prolyl hydroxlases, it retains its activity across a wide range of oxygen tensions (Stolze *et al*, 2004; Dayan *et al*, 2006). This is supported by studies suggesting hypoxic tumours may respond to Bortezomib (Shin *et al*, 2008) and Amphotericin B (Yeo *et al*, 2006), which suppress HIF-1*α* activity by re-enforcing FIH-mediated inhibition of p300 recruitment.

## Figures and Tables

**Table 1:** Flow of familial breast cancer patients through the study, according to REMARK criteria (McShane *et al*, 2005)

	**BRCA1**	**BRCA2**	**BRCAX**	**Total**
Female patients collected for study	45	36	66	147
Patients with tissue available	42	33	63	138
Tumours present on array	38	33	54	125
Tumours with tissue on array and survival data	34	30	52	116

**Table 2:** Clinical and tumour characteristics (*n*=125)

	**BRCA1, n (%)**	**BRCA2, n (%)**	**BRCAX, n (%)**	**All familial, n (%)**
*Age*				
*Median (range), years*				
< 40 years	16 (42%)	9 (27%)	8 (15%)	33 (26%)
40–55 years	20 (53%)	13 (40%)	29 (54%)	62 (50%)
55–69 years	2 (5%)	9 (27%)	14 (26%)	25 (20%)
>70 years	0	2 (6%)	3 (5%)	5 (4%)
				
*Tumour size*				
< 20 mm	29 (76%)	16 (52%)	25 (53%)	68 (60%)
> 20 mm	9 (24%)	15 (48%)	22 (47%)	46 (40%)
Unknown	0	2	7	9
				
*Nodal status*				
Negative	35 (92%)	23 (74%)	30 (63%)	88 (76%)
Positive	3 (8%)	8 (26%)	17 (37%)	28 (24%)
Unknown	0	2	7	9
				
*Grade*				
I	0	1 (4%)	6 (13%)	7 (7%)
II	2 (6%)	14 (48%)	14 (31%)	30 (29%)
III	30 (94%)	14 (48%)	25 (56%)	67 (64%)
Unknown	6	4	9	19
				
*ER-α*				
Negative	29 (85%)	5 (17%)	15 (31%)	49 (44%)
Positive	5 (15%)	24 (83%)	33 (69%)	62 (56%)
Unknown	4	4	6	14
				
*PgR*				
Negative	27 (84%)	10 (35%)	23 (48%)	60 (55%)
Positive	5 (16%)	19 (65%)	25 (52%)	49 (45%)
Unknown	6	4	6	16
				
*HER2 status*				
Negative	29 (100%)	25 (100%)	38 (87%)	92 (93%)
Positive	0	0	7 (13%)	7 (7%)
Unknown	9	8	8	25
				
*Endocrine therapy*				
Not given	28 (90%)	19 (70%)	28 (61%)	75 (72%)
Given	3 (10%)	8 (30%)	18 (39%)	29 (28%)
Unknown	7	6	8	21
				
*Chemotherapy*				
Not given	14 (38%)	17 (52%)	20 (43%)	51 (45%)
Given	23 (62%)	14 (48%)	26 (57%)	63 (55%)
Unknown	1	2	8	11

**Table 3:** Expression of hypoxia markers stratified by familial subtype

	**BRCA1 n (%)**	**BRCA2 n (%)**	**BRCAX n (%)**	**Total n(%)**	***P*-value**
*HIF-1α*					
Negative	9 (28)	20 (63)	29 (59)	58 (51)	0.008
Positive	23 (72)	12 (38)	20 (41)	55 (49)	
					
*PHD1 cytoplasmic*					
Negative	19 (68)	9 (39)	14 (48)	42 (53)	0.105
Positive	9 (32)	14 (61)	15 (52)	38 (47)	
					
*PHD2 cytoplasmic*					
Negative	6 (17)	3 (9)	6 (12)	15 (12)	0.615
Positive	30 (83)	30 (91)	46 (88)	106 (88)	
					
*PHD3 cytoplasmic*					
Negative	20 (53)	10 (30)	15 (28)	45 (36)	0.037
Positive	18 (47)	23 (70)	39 (72)	80 (64)	
					
*PHD1 nuclear*					
Negative	28 (100)	23 (100)	29 (100)	80 (100)	NA
Positive	0	0	0	0	
					
*PHD2 nuclear*					
Negative	34 (94)	27 (81)	44(84)	105 (88)	0.309
Positive	2 (6)	6 (19)	8 (16)	16 (12)	
					
*PHD3 nuclear*					
Negative	32 (85)	25 (77)	36 (67)	93 (74)	0.179
Positive	6 (15	8 (23)	18 (33)	32 (26)	
					
*VEGF*					
Negative	4 (12)	3 (9)	11 (22)	18 (16)	0.228
Positive	31 (88)	30 (91)	40 (78)	101 (84)	
					
*FIH cytoplasmic*					
Negative	19 (53)	10 (32)	27 (53)	56 (47)	0.143
Positive	17 (47)	21 (68)	24 (47)	62 (53)	
					
*FIH nuclear*					
Negative	24 (75)	15 (50)	37 (73)	76 (67)	0.062
Positive	8 (25)	15 (50)	14 (28)	37 (33)	

Abbreviation: NA=not applicable.

**Table 4:** (A) Cytoplasmic PHD3 expression stratified by intrinsic phenotypes in familial breast cancer (*P*=0.037). (B) Nuclear FIH expression stratified by intrinsic phenotypes in familial breast cancer (*P*=0.011). (C) HIF-1α expression stratified by intrinsic phenotypes in familial breast cancer (*P*=0.001)

	**Luminal *n* (%)**	**Basal *n* (%)**	**HER2 *n* (%)**	**Null *n* (%)**	**Total (%)**
*(A)*					
Negative	10 (20)	18 (49)	1 (17)	2 (29)	31 (31)
Positive	39 (80)	19 (51)	5 (83)	5 (71)	68 (69)
Total	49	37	6	7	99
					
*(B)*					
Negative	25 (54)	30 (83)	6 (100)	4 (57)	65 (68)
Positive	21 (46)	6 (17)	(0)	3 (43)	46 (30)
Total	46	36	6	6	95
					
*(C)*					
Negative	33 (70)	9 (25)	4 (67)	3 (50)	49 (52)
Positive	14 (30)	27 (75)	2 (33)	3 (50)	46 (48)
Total	47	36	6	6	95

**Table 5:** Contingency table of hypoxia-induced factors and clinicopathological parameters in familial breast cancer

	**HIF-1α**		**Cytoplasmic PHD1**		**Cytoplasmic PHD2**		**Cytoplasmic PHD3**		**VEGF**		**Cytoplasmic FIH**		**Nuclear FIH**	
	**Neg (%)**	**Pos (%)**	**Neg (%)**	**Pos (%)**	**Neg (%)**	**Pos (%)**	**Neg (%)**	**Pos (%)**	**Neg (%)**	**Pos (%)**	**Neg (%)**	**Pos (%)**	**Neg (%)**	**Pos (%)**
*Size (mm)*														
<20	31 (61%)	33 (61%)	26 (67%)	13 (36%)	9(60%)	57 (59%)	30 (73%)	40 (53%)	13 (81%)	56 (59%)	34 (67%)	29 (50)	43 (59%)	21 (61%)
⩾20	20 (39%)	21 (39%)	13 (33%)	23 (64%)	6 (40%)	40 (41%)	11 (27%)	35 (47%)	3 (19%)	26 (39%)	17 (33%)	29 (50)	30 (41%)	11 (34%)
*P*-value		0.973		0.008		0.928		0.037		0.089		0.079		0.516
														
*Grade*														
1/2	23 (48%)	11 (22%)	9 (25%)	13 (41%)	4 (31%)	32 (36%)	9 (24%)	28 (41%)	3 (25%)	32 (36%)	10 (22%)	22 (41.5)	20 (31%)	13 (42%)
3	25 (52%)	38 (78%)	27 (75%)	19 (59%)	9 (69%)	58 (64%)	28 (76%)	41 (595)	9 (75%)	57 (64%)	36 (78%)	31 (58.5)	45 (69%)	18 (58%)
*P*-value		0.009		0.169		0.735		0.094		0.454		0.036		0.281
														
*ER*														
Neg	14 (26%)	31 (62%)	20 (54%)	12 (35%)	6 (55%)	40 (41%)	22 (58%)	27 (37%)	6 (38%)	38 (43%)	27 (55%)	19 (33.9)	36 (53%)	10 (29%)
Pos	39 (74%)	19 (38%)	17 (46%)	22 (65%)	5 (45%)	57 (59%)	16 (42%)	46 (63%)	10 (62%)	51 (57%)	22 (45%)	37 (66.1)	32 (47%)	24 (71%)
*P*-value		0.001		0.112		0.398		0.035		0.698		0.029		0.024
														
*Lymph nodes*														
Neg	38 (66%)	46 (84%)	35 (83%)	25 (66%)	10 (67%)	81 (76%)	37 (82%)	55 (69%)	44 (80%)	16 (89%)	73 (72%)	44 (71.0)	55 (72%)	30 (82%)
Pos	20 (35%)	9 (16%)	7 (17%)	13 (34%)	5 (33%)	25 (24%)	8 (18%)	25 (31%)	11 (20%)	2 (11%)	28 (28%)	18 (29.0)	21 (28%)	7 (19%)
*P*-value		0.028		0.070		0.413		0.101		0.135		0.344		0.314

Abbreviations: ER=estrogen receptor; Neg=negative; Pos=positive.
