# BRCA1 tumours correlate with a HIF-1*α* phenotype and have a poor prognosis through modulation of hydroxylase enzyme profile expression

**DOI:** 10.1038/sj.bjc.6605430

**Published:** 2009-11-10

**Authors:** M Yan, M Rayoo, E A Takano, KConFab Investigators, S B Fox

**Correction to**: *British Journal of Cancer* (2009) **101**, 1168–1174. doi:10.1038/sj.bjc.6605287

Upon publication of this paper in Volume 101, the group ‘KConFab Investigators’ was incorrectly replaced by H Thorne as co-authors. The authors and publishers are now happy to correct this mistake – the correct authorship is now shown, above.

