# Supplementary figures and images for: BRCA1 tumours correlate with a HIF-1α phenotype and have a poor prognosis through modulation of hydroxylase enzyme profile expression
Source: Br J Cancer. 2009 Sep 1;101(7):1168–74. doi: 10.1038/sj.bjc.6605287 (PMC2768103; doi:10.1038/sj.bjc.6605287)

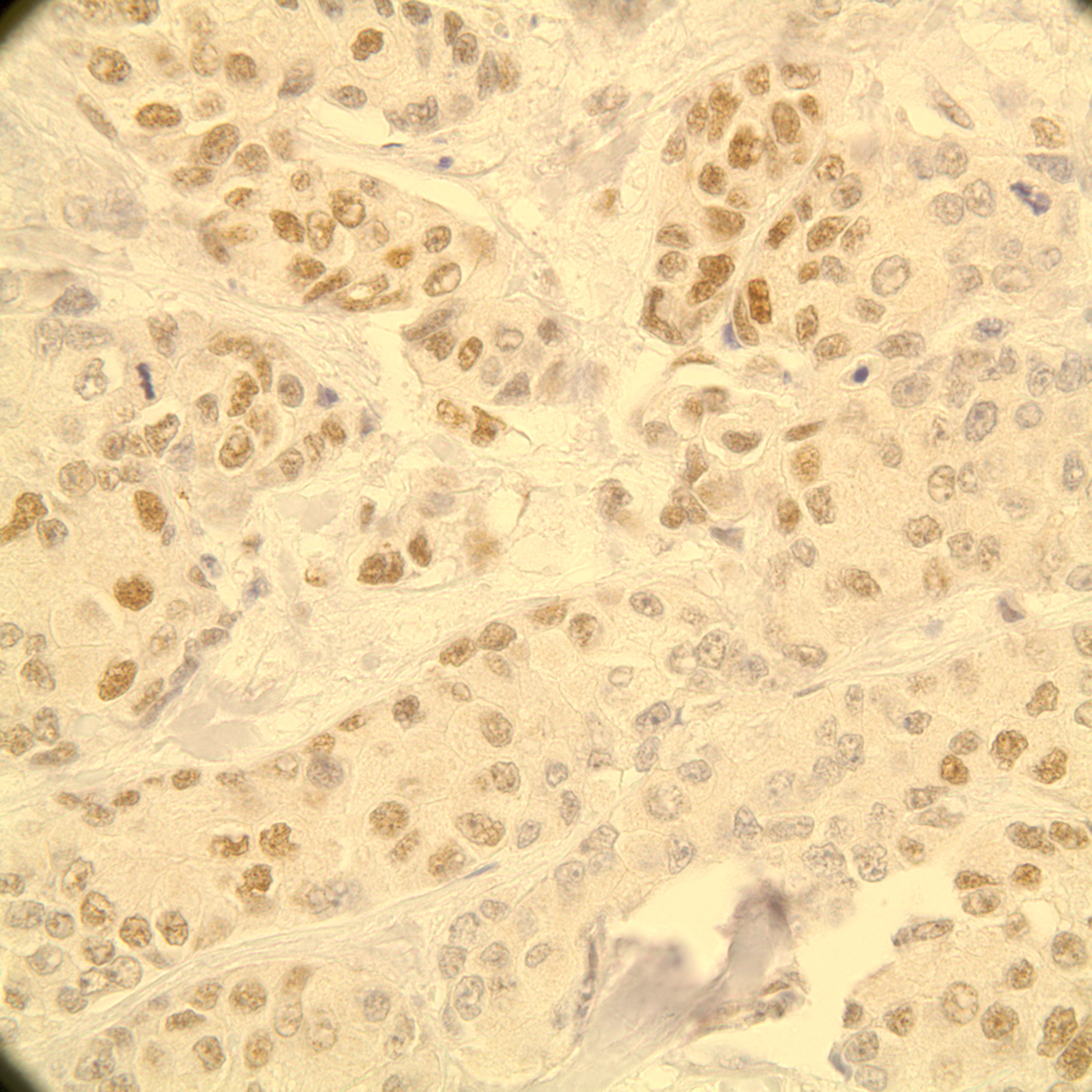

Supplement: Supplementary Figure 1 [file 6605287x1.tif]

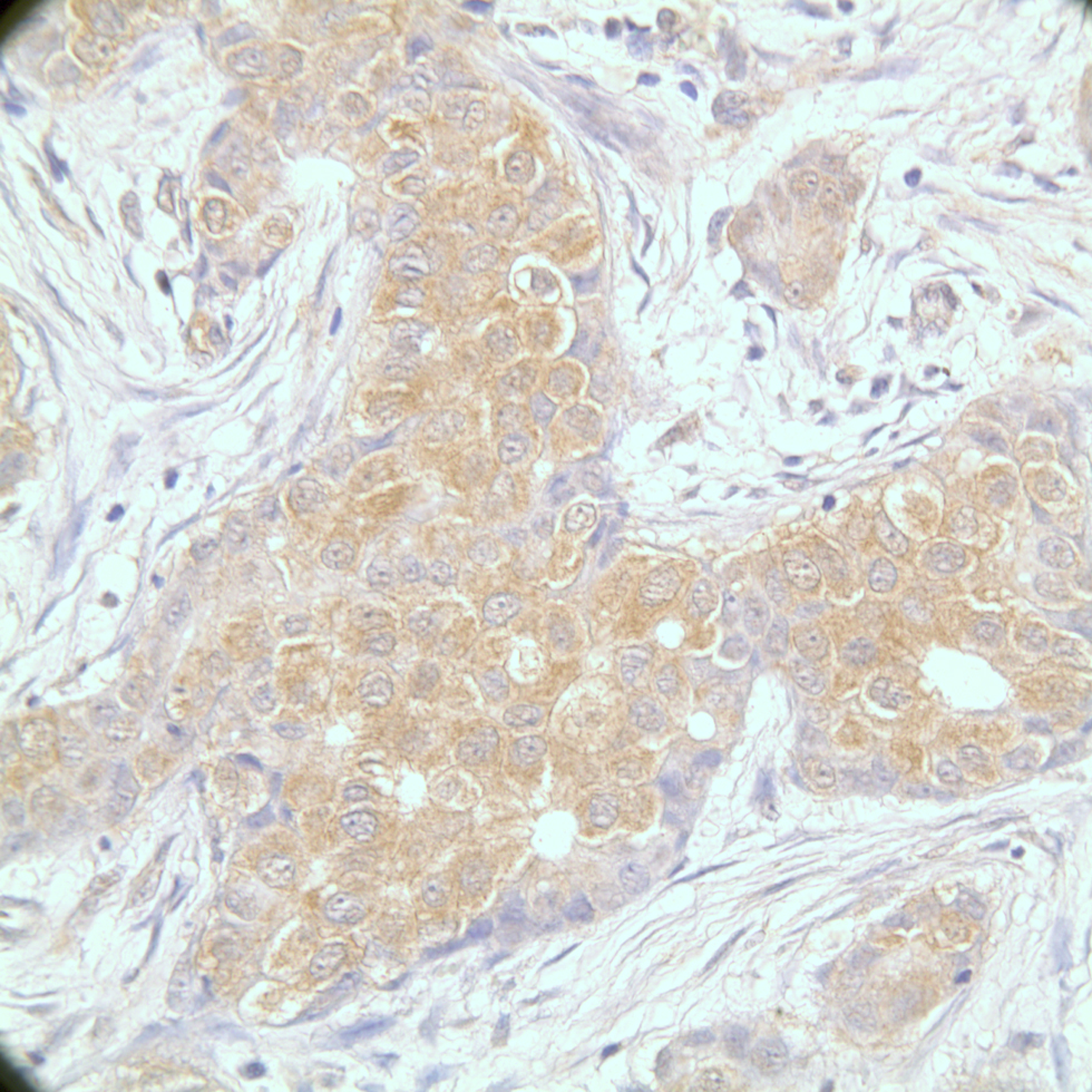

Supplement: Supplementary Figure 2 [file 6605287x2.tif]

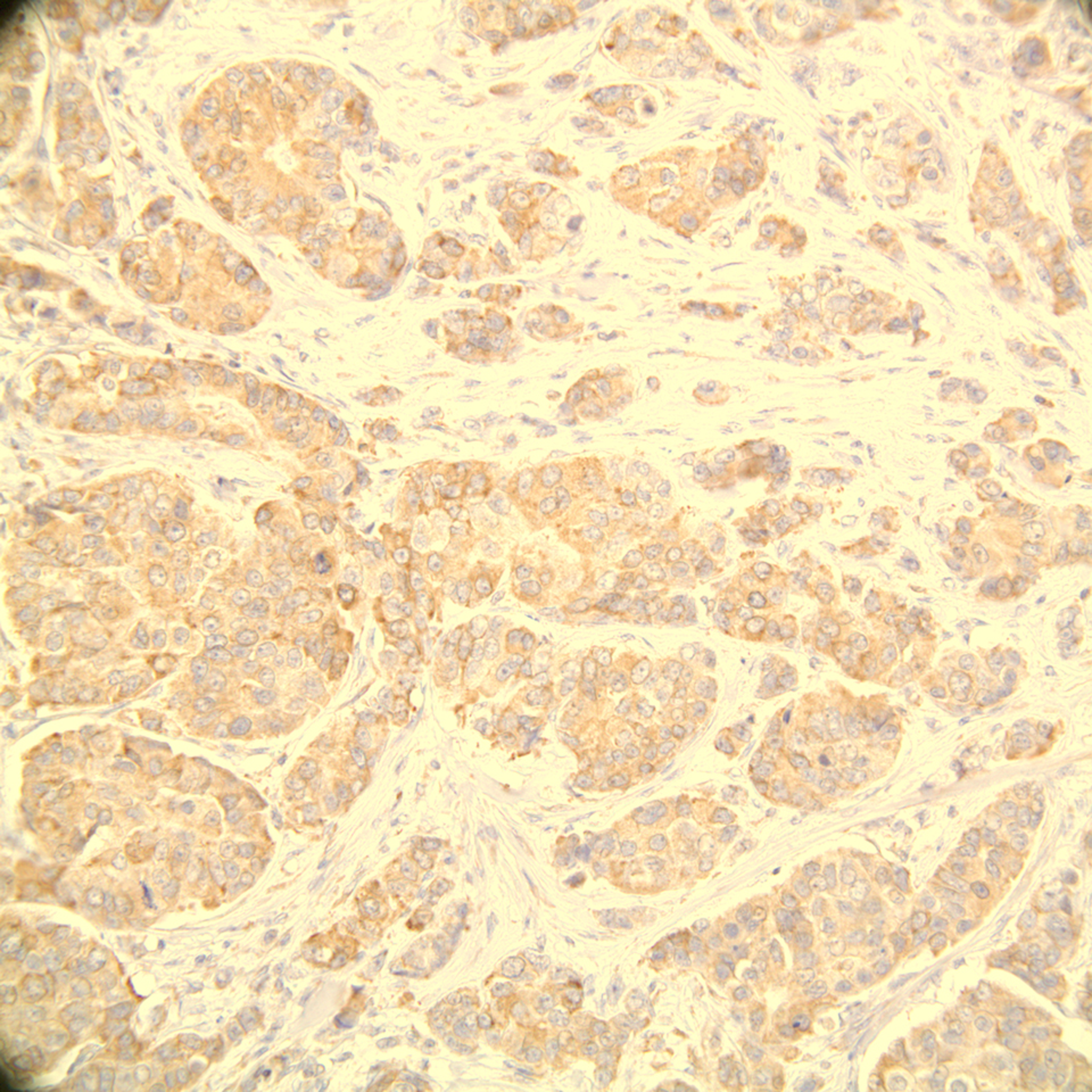

Supplement: Supplementary Figure 3 [file 6605287x3.tif]

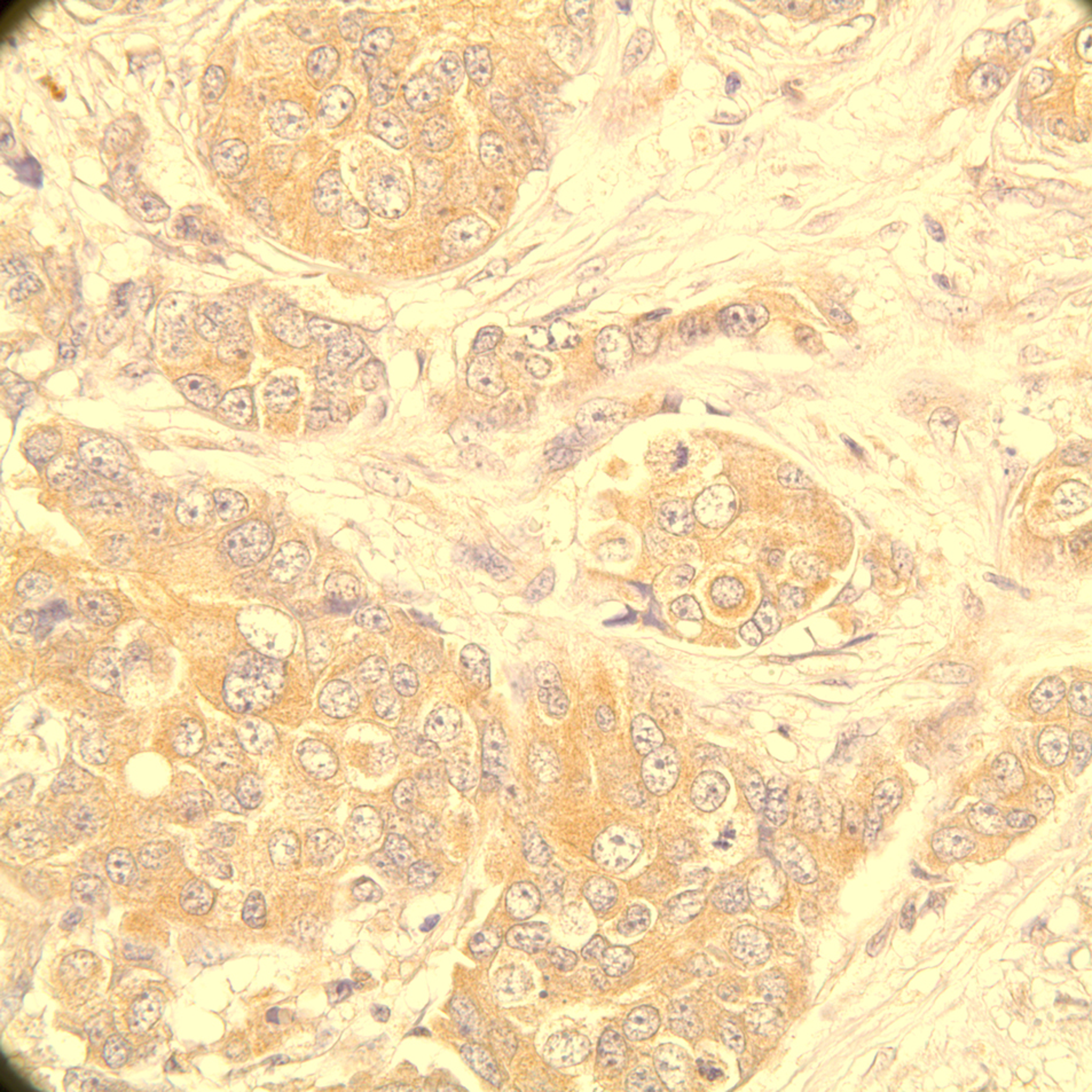

Supplement: Supplementary Figure 4 [file 6605287x4.tif]

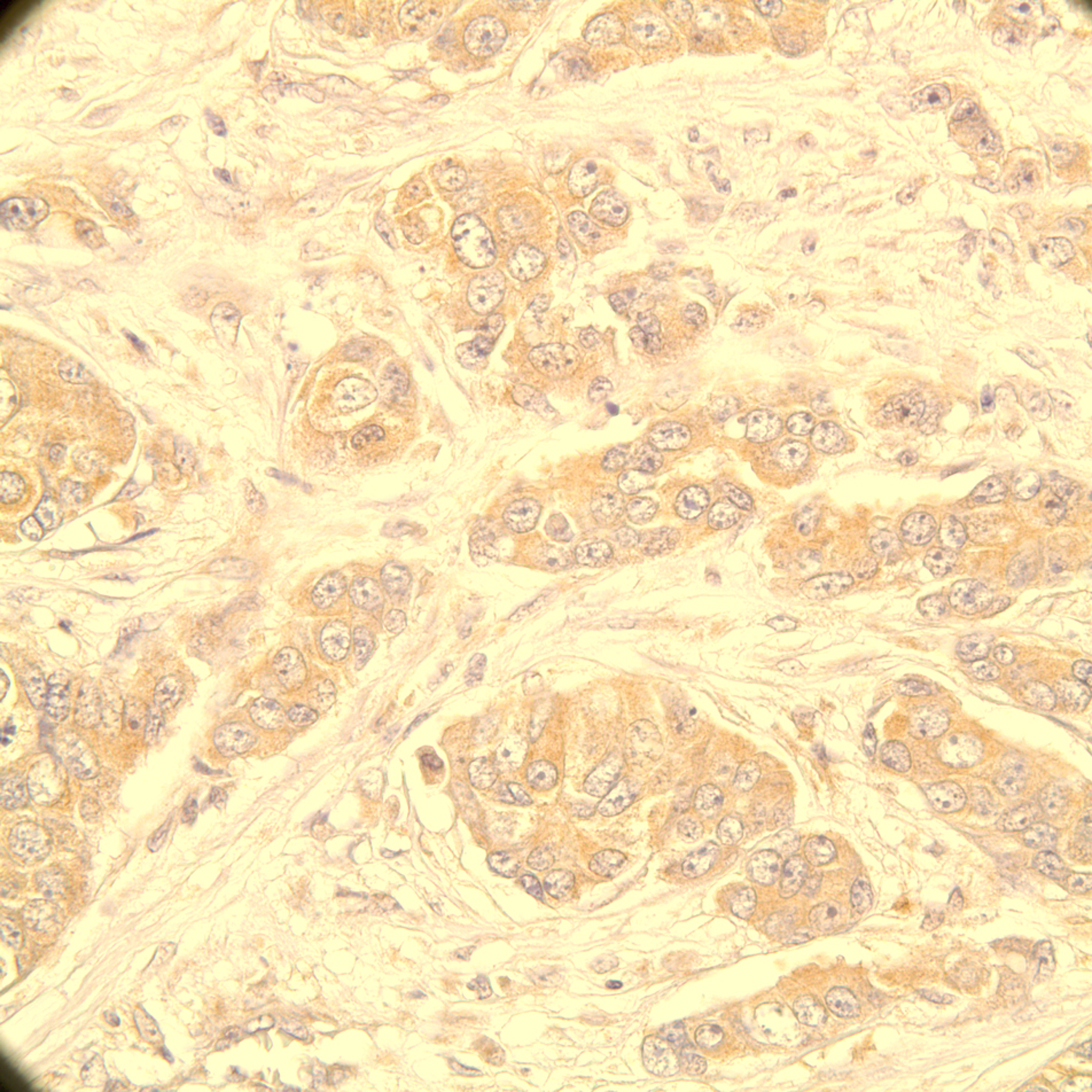

Supplement: Supplementary Figure 5 [file 6605287x5.tif]

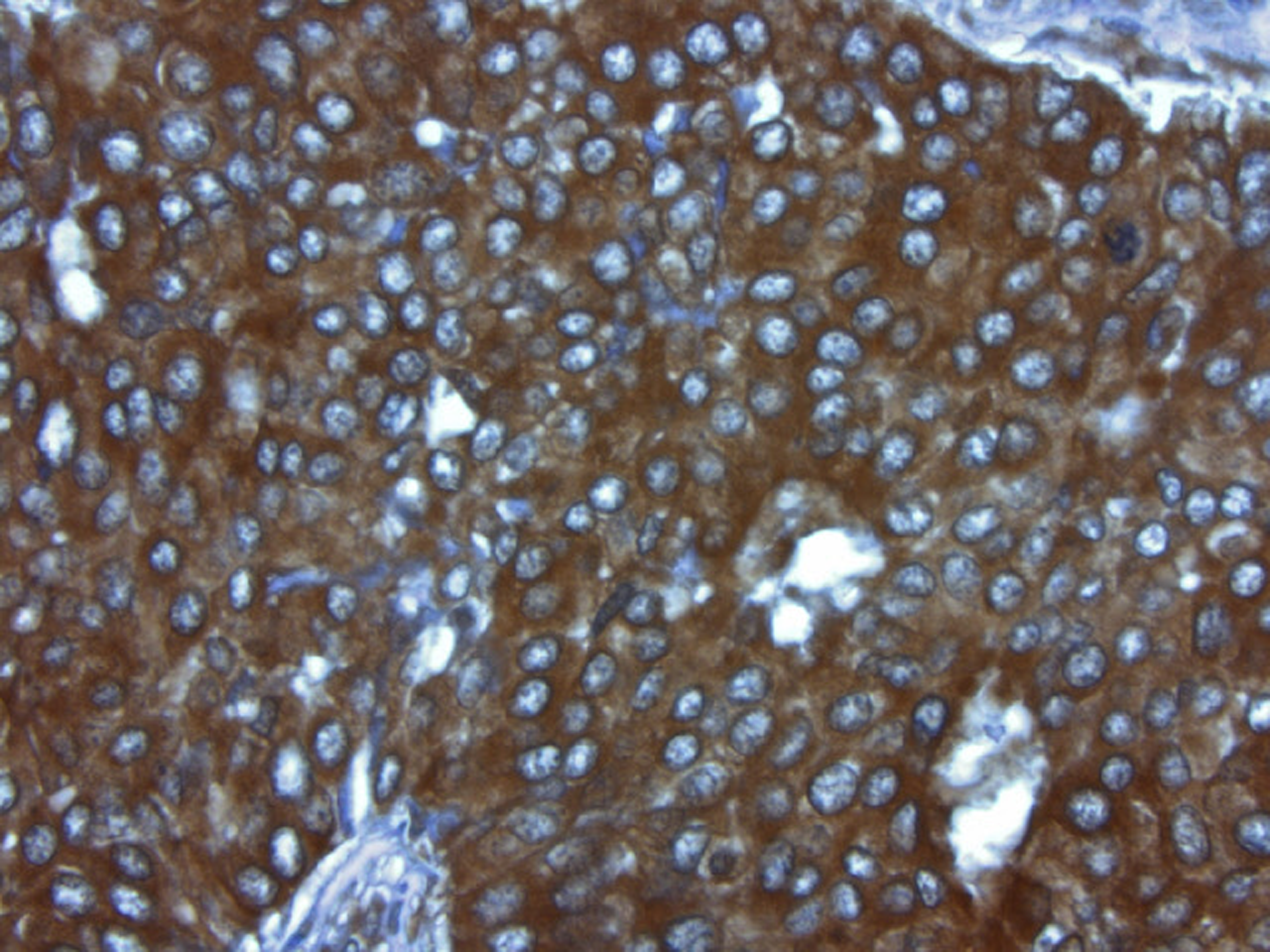

Supplement: Supplementary Figure 6 [file 6605287x6.tif]
